# Author Correction: Soundscapes and deep learning enable tracking biodiversity recovery in tropical forests

**DOI:** 10.1038/s41467-023-42950-8

**Published:** 2023-11-02

**Authors:** Jörg Müller, Oliver Mitesser, H. Martin Schaefer, Sebastian Seibold, Annika Busse, Peter Kriegel, Dominik Rabl, Rudy Gelis, Alejandro Arteaga, Juan Freile, Gabriel Augusto Leite, Tomaz Nascimento de Melo, Jack LeBien, Marconi Campos-Cerqueira, Nico Blüthgen, Constance J. Tremlett, Dennis Böttger, Heike Feldhaar, Nina Grella, Ana Falconí-López, David A. Donoso, Jerome Moriniere, Zuzana Buřivalová

**Affiliations:** 1 Field Station Fabrikschleichach, Department of Animal Ecology and Tropical Biology, Biocenter, University of Würzburg, Glashüttenstr. 5, 96181 Rauhenebrach, Germany; 2 Bavarian Forest National Park, Freyungerstr. 2, 94481 Grafenau, Germany; 3 Fundación Jocotoco, Valladolid N24-414 y Luis Cordero, Quito, Ecuador; 4 Technical University of Munich, School of Life Sciences, Ecosystem Dynamics and Forest Management Research Group, Hans-Carl-von-Carlowitz-Platz 2, 85354 Freising, Germany; 5 Berchtesgaden National Park, Doktorberg 6, Berchtesgaden, 83471 Germany; 6 Saxon-Switzerland National Park, An der Elbe 4, 01814 Bad Schandau, Germany; 7 Yanayacu Research Center, Cosanga, Ecuador; 8 Biodiversity Field Lab (BioFL), Khamai Foundation, Quito, Ecuador; 9 Pasaje El Moro E4-216 y Norberto Salazar, EC 170902 Tumbaco, DMQ Ecuador; 10 Rainforest Connection, Science Department, 440 Cobia Drive, Suite 1902, Katy, TX 77494 USA; 11 Ecological Networks Lab, Department of Biology, Technische Universität Darmstadt, Schnittspahnstr. 3, 64287 Darmstadt, Germany; 12 Phyletisches Museum, Institute for Zoology and Evolutionary Research, Friedrich-Schiller-University Jena, Jena, Germany; 13 Animal Population Ecology, Bayreuth Center for Ecology and Environmental Research (BayCEER), University of Bayreuth, 95440 Bayreuth, Germany; 14 Grupo de Investigación en Biodiversidad, Medio Ambiente y Salud-BIOMAS-Universidad de las Américas, Quito, Ecuador; 15 Departamento de Biología, Facultad de Ciencias, Escuela Politécnica Nacional, Av. Ladrón de Guevara E11-253, CP 17-01-2759 Quito, Ecuador; 16 AIM - Advanced Identification Methods GmbH, Niemeyerstr. 1, 04179 Leipzig, Germany; 17 University of Wisconsin-Madison, Department of Forest and Wildlife Ecology and The Nelson Institute for Environmental Studies, 1630 Linden Drive, Madison, WI 53706 USA

**Keywords:** Conservation biology, Animal behaviour

Correction to: *Nature Communications* 10.1038/s41467-023-41693-w, published online 17 October 2023

In this article, the author name Jack LeBien was incorrectly written as John G LeBien. The original article has been corrected.

