# Supplementary material for: Soundscapes and deep learning enable tracking biodiversity recovery in tropical forests
Source: Nat Commun. 2023 Oct 17;14:6191. doi: 10.1038/s41467-023-41693-w (PMC10582010; doi:10.1038/s41467-023-41693-w)
Supplement: Supplementary file 1 — Supplementary Information File [file 41467_2023_41693_MOESM1_ESM.pdf]

Supplementary material

Soundscapes and deep learning enable tracking biodiversity recovery in tropical forests

Jörg Müller, Oliver Mitesser, H. Martin Schaefer, Sebastian Seibold, Annika Busse, Peter Kriegel, Dominik Rabl, Rudy Gelis, Alejandro Arteaga, Juan Freile, Gabriel Augusto Leite, Tomaz Nascimento de Melo, John G. LeBien, Marconi Campos-Cerqueira, Nico Blüthgen, Constance J. Tremlett, Dennis Böttger, Heike Feldhaar, Nina Grella, Ana Falconí-López, David A. Donoso, Jerome Moriniere, Zuzana Buřivalová

Sound analyses

Selection of audio files for expert identification

Experts listened to soundscapes and looked at the spectrogram, they were allowed to re-listen as many times as they wanted. They were blinded to the spatial location and habitat type of the audio file. Unknown amphibian species were treated as sound-morpho-species. All amphibian species were anonymized for conservation reasons.

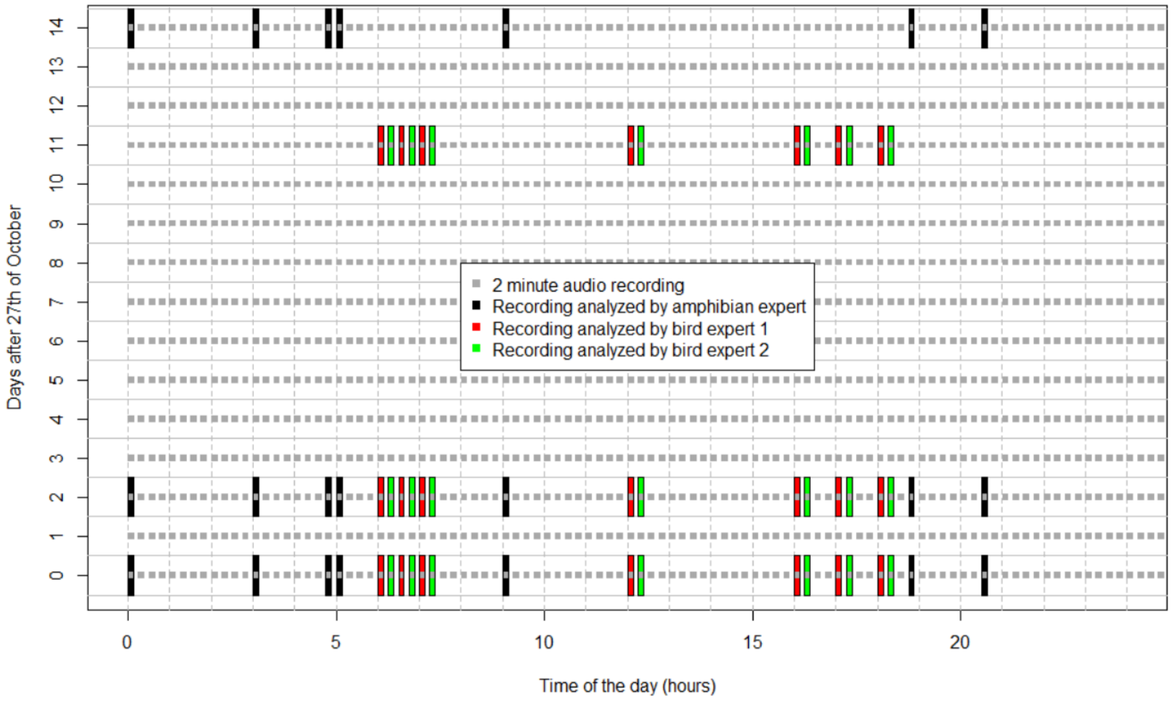

Figure S1: Overview on the time windows selected for expert identification from all sampled audio files during two weeks.

Table S1: List of 77 bird species selected from CNN models with probability to occur in our study site Canande, based on a local species list. From these species four were further excluded either because of low probability of occurrence in the study area and/or because of no detection via CNN model. Shared species occurred in the data set of CNN identification and in the data identified by expert 1.

Bird Species	Unprobable	Not recorded in CNN with prob >0.8	Shared 49 species
Aburria aburri			x
Amazona autumnalis			
Ara ambigua			
Campephilus gayaquilensis			x
Camptostoma obsoletum			x
Campylorhamphus trochilirostris		x	
Cantorchilus leucopogon			x
Celeus loricatus			x
Claravis pretiosa			x
Clibanornis rubiginosus			x
Colaptes rubiginosus			
Conopias albovittatus			x
Contopus sordidulus			x
Cryptoleucopteryx plumbea			
Crypturellus soui			x
Cyanoloxia cyanoides			
Cyclarhis nigrirostris	x		
Dendrocincla fuliginosa			x
Dendrocolaptes sanctithomae			x
Dendrocygna autumnalis	x	x	
Dryobates callonotus			
Dysithamnus puncticeps			x
Elaenia flavogaster			x
Electron platyrhynchum			x
Formicarius nigricapillus			x
Furnarius leucopus			x
Glaucidium peruanum			
Grallaria guatimalensis			x
Gymnopithys bicolor			
Hafferia zeledoni			x
Hylopezus perspicillatus			x
Legatus leucophaius			x
Lepidocolaptes souleyetii			x
Leptotila verreauxi			x
Lipaugus unirufus			x
Lophostrix cristata			
Lophotriccus pileatus			x
Micrastur semitorquatus			

Microbates cinereiventris		x
Microcerculus marginatus		x
Momotus subrufescens		
Notharchus hyperrhynchus		
Nyctidromus albicollis		
Nyctiphrynus rosenbergi		x
Nystalus radiatus		x
Ornithion brunneicapillus		x
Ortalis erythroptera		
Patagioenas subvinacea		x
Penelope purpurascens		x
Phaenostictus mcleannani		x
Pharomachrus auriceps		
Philydor rufum		
Piaya cayana		x
Poliocrania exsul		
Pseudastur occidentalis		
Querula purpurata		x
Ramphastos ambiguus		x
Ramphastos brevis		x
Ramphocaenus melanurus		x
Saltator atripennis		
Saltator grossus		x
Saltator maximus	x	
Schiffornis veraepacis		x
Sipia nigricauda		
Sittasomus griseicapillus		
Spizaetus ornatus		
Sporophila funerea		x
Taraba major		x
Thamnistes anabatinus		
Tinamus major		x
Trogon collaris		x
Trogon comptus		x
Trogon mesurus		
Trogon rufus		x
Turdus maculirostris		x
Xiphorhynchus erythropygius		x
Zimmerius albigularis		x

Species richness across habitat types

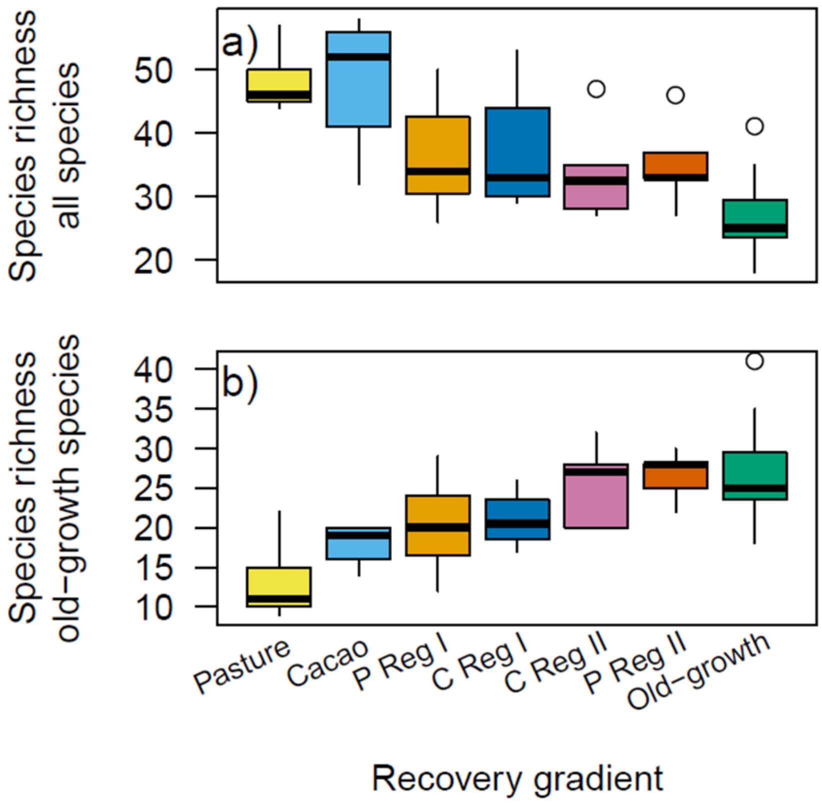

Figure S2: Richness values across recovery stages. (a) Total species richness of vocalizing vertebrates and (b) richness of vocalizing vertebrate species observed in our old-growth plots. For agricultural legacies (P = pasture, C = cacao) duration of recovery ranges from 1-19 years (Reg I) and 20-34 years (Reg II). Each of the seven categories is displayed in different colours.

Comparison of two bird experts

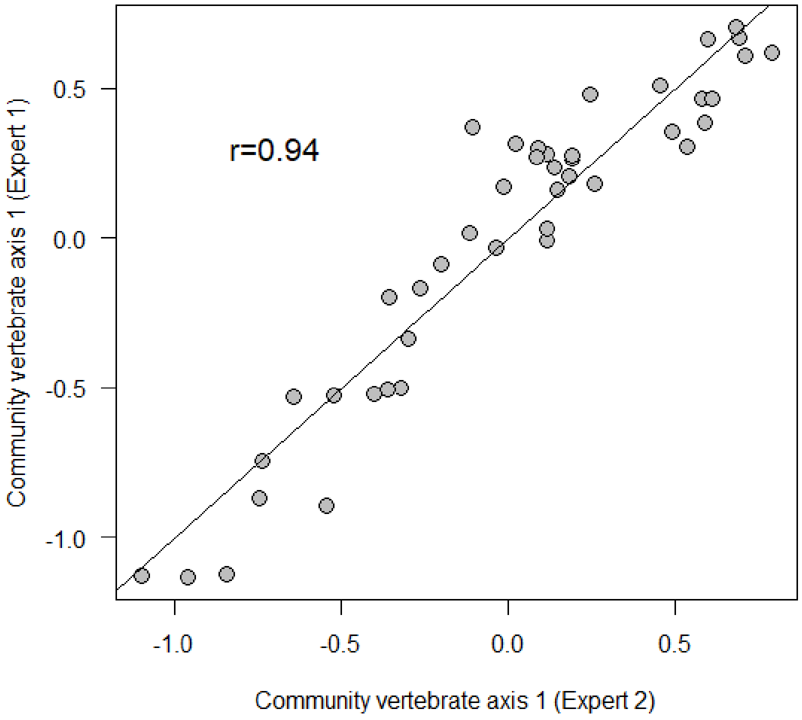

Figure S3: Comparison of NMDS community axis 1 based on two data sets with identification of bird species by two different bird experts.

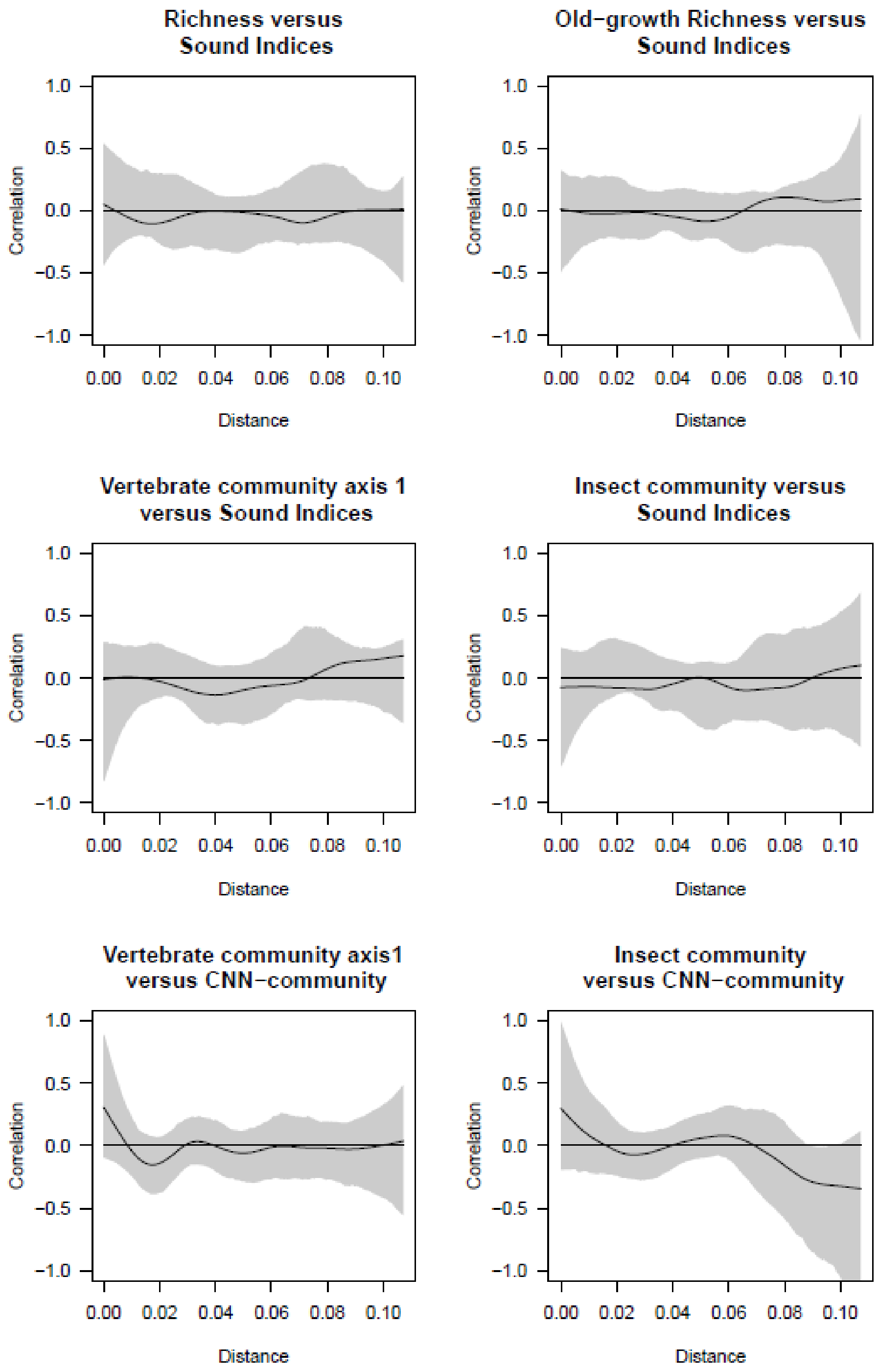

Figure S4: Spline correlogram plots to test for spatial independence of model residuals of the 4 models using acoustic indices (upper two rows) and CCN-based community axis 1 (lower row) as predictor. In all plots the 95% pointwise grey confidence band includes the zero line, underlining spatial independence of residuals in our models.
